# Personalized medicine in Parkinson's disease: Time to be precise

**DOI:** 10.1002/mds.27027

**Published:** 2017-06-12

**Authors:** Nataliya Titova, K. Ray Chaudhuri

**Affiliations:** ^1^ Federal State Budgetary Educational Institution of Higher Education “N.I. Pirogov Russian National Research Medical University” of the Ministry of Healthcare of the Russian Federation Moscow Russia; ^2^ National Parkinson Foundation International Centre of Excellence, King's College London and King's College Hospital London UK; ^3^ Department of Basic and Clinical Neuroscience The Maurice Wohl Clinical Neuroscience Institute, King's College London London UK; ^4^ National Institute for Health Research South London and Maudsley NHS Foundation Trust and King's College London London UK

The treatment of PD remains underpinned by levodopa and other dopamine replacement therapies (DRT). Although DRT and in particular levodopa has the ability to improve the motor symptoms of PD, motor complications still bedevil the treatment strategies in PD. In addition, new challenges have emerged. PD is now recognized as a multisystem, multineurotransmitter dysfunction‐related heterogeneous disorder.^1^, ^2^ Biomarker‐driven evidence suggests that PD is a complex disease that could present with nondopaminergic syndromes.^2^ Characteristics of these patients with nonmotor subtypes have been recently described.^3^, ^4^ Therefore, in many, the generic prescribing of DRT may not be sufficient, and we need to be aware of the “one size does not fit all” concept regarding treatment. Consideration of specific personal needs and the clinical phenotype of patients before prescribing is the basis of personalized medicine. The definition of personalized medicine is variable, and the American Medical Association has defined this as “health care that is informed by each person's unique clinical, genetic, and environmental information.” Personalized medicine is an important consideration for “single multifactorial” pathology‐driven conditions and may require the use of “cocktail therapies.” This concept is now particularly relevant for PD given the multiple pathology culminating in a complex motor and nonmotor disorder.^2^ In PD, for example, treatment needs to be prescribed based on the susceptibility of specific subtypes of PD to side effects (subtype‐specific treatment) or consideration of lifestyle, genetic framework, personality, and pharmacogenetics. This concept of personalized medicine in PD is relatively new, and the defining enablers of this strategy are shown in Figure 1. We accept that there may be substantial overlap between some components such as genetics versus pharmacogenetics or aging with comorbidity. However, true individualization of treatment needs to take into account these factors separately. Personalized medicine can also comprise of various substrategies ranging from a holistic concept to precision medicine based on genomics (Fig. 2). In this article, we consider the various concepts that may help development of functionally effective personalized medicine in PD.

**Figure 1:**
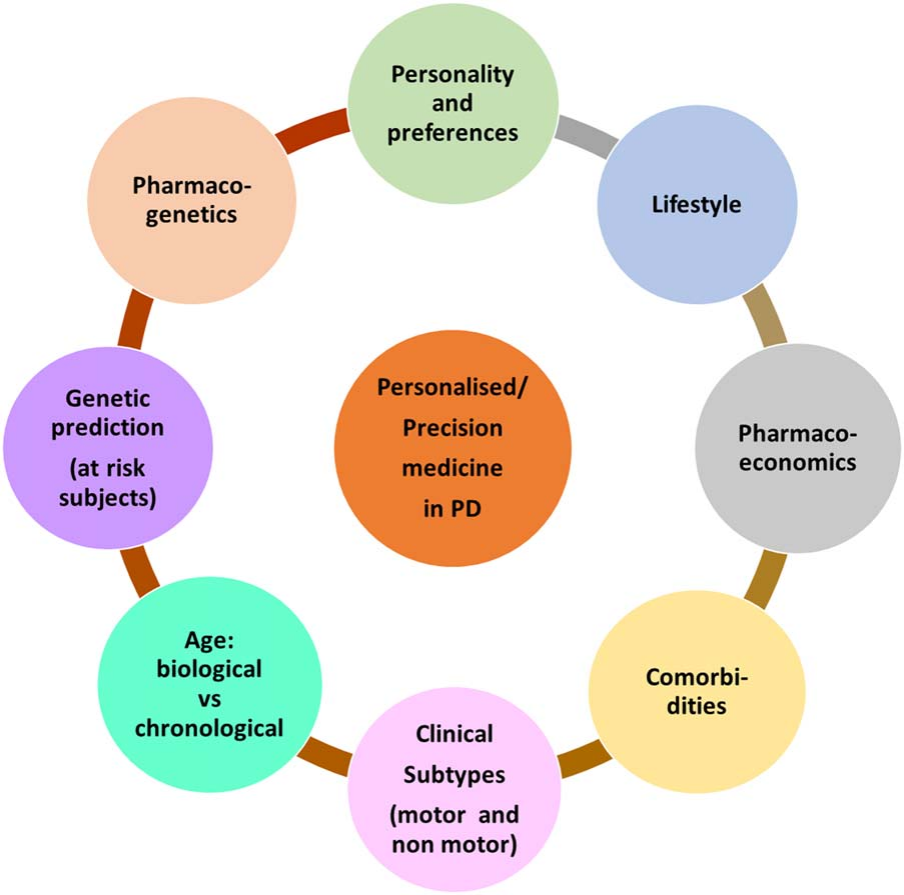
A diagram of potential factors to consider which may drive or enable pathways for personalized and precision medicine in PD.

**Figure 2:**
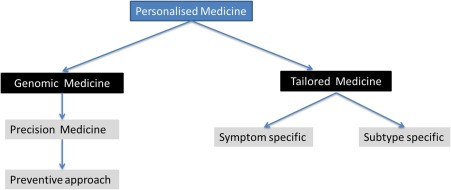
Proposed types of personalized medicine applicable to PD. [Color figure can be viewed at wileyonlinelibrary.com]

## Genetics and Pharmacogenetics

**Figure 3:**
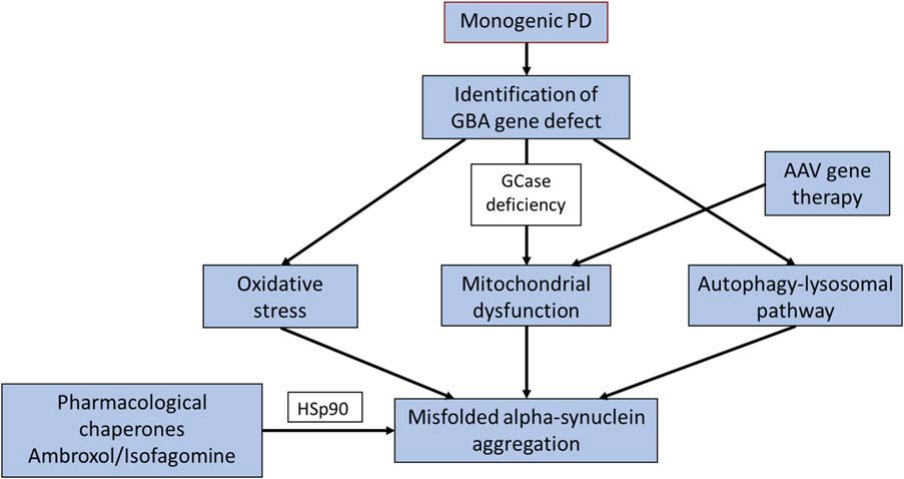
An example of how precision medicine based “cocktail” therapy could be applied in the context of genomic personalized medicine in patients carrying the glucocerebrocidase mutation in PD. *GBA*, glucocerebrocidase gene; GCase, glucocerebrocidase; AAV, adeno‐associated virus. [Color figure can be viewed at wileyonlinelibrary.com]

Personalized medicine could predict the susceptibility for the development of PD in an individual basis, and the genetics of PD is important in this context. Identifying at‐risk individuals through known genetic susceptibility markers in the preprodromal stage of PD could help precision medicine delay or stop the development of clinical PD (Fig. 3).^5^ Although genome‐wide association studies have identified a number of PD loci, these do not explain the main bulk of heritability issues in PD. Monogenic PD is rare; however, early‐onset autosomal dominant presentations can identify specific genes (such as mitochondrial genes *DJ‐1*, Phosphatase and tensin homolog (PTEN)‐induced kinase 1 (*PINK1*)) or gene products (aberrant oligomeric alpha‐synuclein aggregates). Potentially, this knowledge would identify mechanisms resulting in the mishandling of alpha‐synuclein and the formation of aberrant oligomeric aggregates (Fig. 3). Specific therapies can then be developed to counteract these mechanisms.

One example is the increased frequency of PD in heterozygote carriers of the glucocerebrocidase gene (*GBA*), and approximately 5% to 10% of PD patients have *GBA* mutations.^6^ A *GBA* mutation is currently the most important genetic predisposing risk factor for PD, particularly in the White population, although there are racial variations. For instance, the GBA genotype at rs6812193 single nucleotide polymorphism is not seen in the Chinese population.^7^ There is a reciprocal relationship between glucocerebrocidase (*GCase*) activity and alpha‐synuclein function.^8^ Enhancing GCase activity may lead to regulation or even attenuation of the formation of misfolded oligomeric alpha‐synuclein.^8^ In a mouse model of Gaucher's disease, adeno‐associated viral vector delivery of the recombinant GCase into the brain caused modulation of alpha‐synuclein deposition and improved memory deficits.^6^, ^9^ Precision medicine strategy could also be driven by the alteration of activity of chaperone protein such as Hsp90 involved in recognition of misfolded alpha‐synuclein (Fig. 3). Specific histone deacetylase or Hsp90 inhibitors acting as pharmacological chaperones such as ambroxol or isofagomine may therefore be beneficial, and clinical trials are in progress.^6^, ^8^ In terms of personalized medicine, a strategy of combined chaperone and GCase augmentation‐based “cocktail” therapy could be useful for GBA‐positive carriers who remain at risk of conversion to clinical PD. This method could also be useful in those with sporadic PD and documented reductions in GCase activity (Fig. 3). This is particularly relevant as *GBA* gene mutation variants have been shown to be associated with a specific cognitive subtype in PD with a rapid cognitive decline progressing to dementia.^9^ Similarly, the identification of the carriers of the leucine‐rich repeat kinase 2 gene (*LRRK2*) could be targeted with *LRRK2* inhibitors. In rodent models, the *LRRK2* inhibitor GNE‐7915 enhanced the release of dopamine and also synaptic vesicle mobilization and recycling.^10^

Another approach of personalised medicine is pharmacogenetics. Pharmacogenetics implies the influence of inherited genetic differences in drug metabolic pathways which affect individual clinical responses to drugs as well as adverse events.^11^, ^12^ In PD, the role of pharmacogenetics is slowly evolving and some examples include the following:

- The mutations of the catechol‐O‐methyl transferase (*COMT*) gene and response to levodopa based on high‐ and low‐activity alleles,^13^
- The genetic mutations associated with impulse control disorders (dopamine receptor D3 (DRD3) [AA genotype], glutamate ionotropic receptor NMDA type subunit 2B (GRIN2B) [CC geneotype], HTR2A c.102T allele),^14^, ^15^
- Dopamine receptor D2 (DRD2) (CA dinucleotide short tandem repeat) polymorphism appearing to show a protective effect on development of levodopa‐induced dyskinesias in men but not in women.^16^

In addition, single nucleotide polymorphisms rs2283265 and rs1076560 of the *DRD2* gene have been reported to be significantly associated with a good response to rasagiline in early PD.^17^ Possible clinical implications are outlined in Table 1; however, it must be pointed out that there are contradicting studies, and at this moment no definitive recommendations can be provided.^12^ Pharmacogenetic strategies could also potentially be useful for excessive daytime sleepiness (COMT polymorphism, DRD2, and DRD4 [both linked to “sleep attacks”]), hypocretin neuropeptide precursor (HCRT) (prepro‐hypocretin), and psychosis (dopamine receptor D4 (DRD4), cholecystokinin (CCK), apolipoprotein E (APOE4), angiotensin convertin enzyme (ACE)) in PD, although clinical implications are unclear and controversial.^18^, ^19^

**Table 1:** The application of personalised medicine strategy based on pharmacogenomic factors and clinical presentation^17^, ^18^, ^19^

The clinical symptom	The gene/genotype	Clinical effect	Proposed personalized medicine strategy
Levodopa response	COMT: Val158Met (rs4680): low activity COMT (Val/Val): high activity COMT (Val/Val) high activity SLC6A3 rs3836790 6/6 or rs28363170 10/10	Levodopa accumulation Levodopa hypo‐responsiveness in high‐activity COMT gene Better response to COMT inhibitors Better benefit from levodopa	Caution with levodopa dose 1. Levodopa challenge test in high dose to confirm PD diagnosis 2. Aim for higher dosing of levodopa to produce beneficial effect in carriers of high activity COMT Consider ethnic variations (COMT activity varies in different racial groups) Consider preferential use of COMT inhibitors Consider lower doses of levodopa and longer inter‐dose intervals
ICD expression	DRD3 (AA genotype), GRIN2B (CC genotype), HTR2A c.102T allele	Increased susceptibility to ICD Punding: (HTR2A c.102T allele)	1. Genetic screening could predict where DA needs to be used with caution and close monitoring 2. Use of DA sparing strategies 3. Pretreatment counseling 4. Avoid short acting “rescue” therapy
Levodopa‐nduced dyskinesias	DRD2 (CA_n_‐STR)	Increased dyskinesias in women	1. Monitor and start on levodopa sparing or low dose levodopa strategies

COMT, catechol o methyl transferase; ICD, impulse control disorder; DRD2, dopamine receptor D2; DRD3, dopamine receptor D3; DRD2 (CA_n_‐STR), dopamine receptor D2 (CA dinucleotide short tandem repeat; DA, dopamine agonist; HTR2A, 5‐hydroxytryptamine receptor 2A); GRIN2B (CC genotype), glutamate ionotropic receptor NMDA type subunit 2B; Val, valine; Met, methionine; SLC6A3, dopamine transporter type 1‐encoding gene.

### Age: Biology, Chronology, and Personalized Medicine

Aging is a complex process, and there may be differences between chronological versus biological aging. However, many anti‐PD therapeutic strategies define *age* as a definitive landmark that influences therapy. For instance, in clinical practice dopamine agonists are often not prescribed in “older” PD (defined by chronological age) because of the possibility of side effects; in addition, deep brain stimulation is usually not attempted beyond 65 to 70 years. Such generic strategies do not take into consideration “healthy aging,” a longer life span, and differences between biological and chronological aging. Aging‐related variables that may influence personalized medicine are outlined in Table 2.

**Table 2:** Factors that may influence personalized and precision medicine strategies related to age (young and old)

• Genetic: telomeres and telomere length as a possible biomarker of biological aging
• Comorbidity: whether present or absent (see Table 3)
• Imaging biomarkers: magnetic resonance imaging
• DRT‐related adverse events (ICD, dyskinesias) in younger patients
• Tolerability of DRT (young vs old)

DRT, dopamine replacement therapy; ICD, impulse control disorder.

Telomeres are crucial for adjusting cellular response to stress as well as the stimulation of cell growth and work by “capping” chromosomes (Table 2).^20^ With the accumulation of “uncapped” or short telomeres, apoptosis and cell death are triggered. Aging is associated with a decline in telomere length that results in a progressive functional reduction of tissue function and causes mortality although studies have suggested that short telomere may not be linked to PD.^20^ Clinically, enhancing telomerase functional activity as well as the inhibition of telomerase activity have been explored in cancer therapy, and a telomerase template unit (GRN163L) is currently undergoing clinical trials.^21^ Telomerase immortalized midbrain astrocytes has been used in rodent PD models to direct stem cells to dopaminergic cells.^22^ Although there was dopaminergic neurogenesis, there was also uncontrolled expansion similar to tumorogenesis.

Personalized medicine concurrent with aging could be supported by magnetic resonance imaging showing focal (medial temporal or global) atrophy or white matter vascular disease, suggesting an increased propensity to cognitive impairment. This should trigger early consideration for cognitive testing and a low threshold for the use of cholinesterase inhibitors in addition to social and home care support.

Some dopamine agonists such as transdermal rotigotine patch are well tolerated in older patients and maybe a suitable and preferable choice, particularly if there are gastrointestinal issues.^23^, ^24^

### Comorbidities

PD is associated with a number of comorbidities that may guide management strategy of PD independent of aging (Table 3). Examples include type 1 diabetes in younger PD versus type 2 in older patients, whereas thyroid dysfunction can cross any age group. Personalized medicine strategies should thus take into account the impact of cerebrovascular and cardiovascular risk factors (some antihypertensives have a dopamine‐blocking effect), the influence of diabetes and osteoporosis, a major problem in older female patients with PD.

**Table 3:** Comorbidity in PD and proposed personalized medicine strategy

Systems involved	Potential risks	Strategies
Cerebrovascular	Risk of vascular dementia and stroke/TIA Risk of severe hyperhomocysteinaemia in those on levodopa therapy	Lifestyle advise Vascular risk factor management Monitor plasma homocysteine levels in those with high dose levodopa therapy Potential role of checking MTHFR C677T polymorphism of the MTHFR gene in severe hyperhomocysteinaemia^18^
Cardiovascular	Risk of cardiac dysrhythmia with prolonged QTc interval	Avoid drugs prolonging QTc interval (eg, antipsychotics such as quetiapine)
Endocrine	**Thyroid**: **Hypothyroid** Apathy, depression **Hyperthyroid**: Weight loss, anxiety **Testosterone deficiency**: Depression, anxiety, fatigue, decreased libido, sexual dysfunction **Diabetes**: Unclear association.^25^ Often associated with gastroparesis, postural hypotension, urinary dysfunction, diarrhoea, and erectile dysfunction	Metabolic screening in patients with relevant non motor symptoms (depression, apathy, anxiety, weight loss) Endocrine referral and relevant management of thyroid disorder Role of testosterone supplementation is unclear Ensure good diabetic control and management of autonomic dysfunction (also covered under nonmotor subtype section)
Bone health (osteoporosis)	High prevalence with median age of 75 years; high risk of hip fracture	Active, primary, and secondary prevention in all older PD patients with vitamin D2 and biphosphonates^26^ Physiotherapy to reduce fear of falling and better gait strategy
Weight	Weight loss may be: A specific phenotype^27^ with high risk of dyskinesias Secondary to gastrointestinal dysfunction (dysphagia, malabsorption) Secondary to hyperthyroidism	In those with low body weight: Consider lower levodopa dosing, nutritional supplements Nonoral therapies in those with proven GIT disturbances

MTHFR, methylenetetrahydrofolate reductase; TIA, transient ischaemic attacks; GIT, gastrointestinal tract.

### Personality and Perception of Treatment (Listening to the Patient)

Patient choice and informed decision making is key to the 21st‐century management of PD. The main factors related to personality and personalized medicine are listed in Table 4. Successful treatment of PD should consider personality traits that may be a risk factor for the development of impulse control disorders (ICD), dopamine dysregulation syndrome, and levodopaphobia.^28^, ^29^, ^30^ Personal and cultural beliefs such as a reliance on complementary or alternative therapies could inherently make the patient less likely to accept conventional DRT. In some patients, rigid perceptions may influence the acceptability of the DRT delivery pattern. As an example, some patients may find nonoral therapies unacceptable. Personalized medicine strategy in these patients should include close liaison with primary and secondary care in addition to detailed explanations of DRT. Those who have had poor compliance with multiple dosing–based previous treatment strategies or are currently noncompliant for DRT, need to be considered for once‐a‐day therapy.

**Table 4:** Personality trends and attitudes toward treatment that may affect personalized medicine in Parkinson's disease

Personality predisposing to reward seeking behavior and risk factors for ICD, dopamne dysregulation, and punding 28, 29, 30 Novelty seeking behavior High alcohol consumption History of substance abuse and drug addiction Single status
Presence of medical therapy phobia, particularly levodopaphobia
Belief in alternative or complementary therapies such as homeopathy
Personal and cultural belief of patient and carergiver
Past and current history of noncompliance to regular prescribing

ICD, impulse control disorder.

### Lifestyle

**Figure 4:**
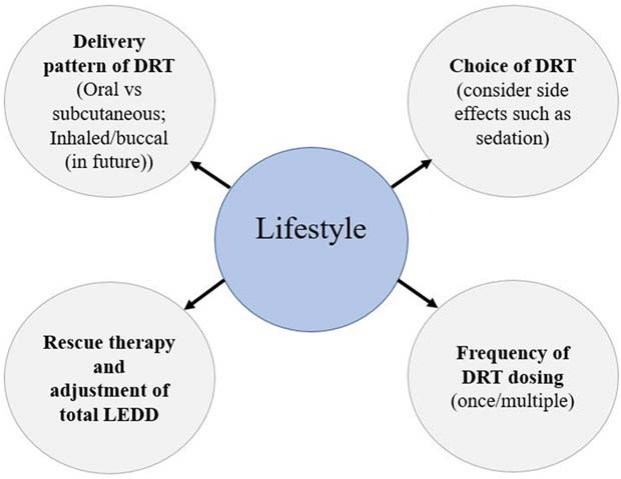
A circle of components of lifestyle that will help complete delivery of personalized medicine. [Color figure can be viewed at wileyonlinelibrary.com]

Activity levels related to lifestyle choice are important because patients active in sport and profession may prefer once‐a‐day therapy as opposed to DRT taken several times a day (Fig. 4). The delivery pattern of dopaminergic drugs may also be relevant (oral vs nonoral). Concern over the loss of employment and the type of employment could influence personalized medicine. In an employed younger PD patient, one may have to opt for rapid relief of motor and nonmotor symptoms by using appropriate DRT or rescue therapies so that the patient can continue to work. In others engaged in machinery operating or work requiring high levels of alertness, sedating DRT and other therapies need to be avoided.

### Pharmacoeconomics

The acceptability of prescribed DRT in PD depends on the affordability and the local reimbursement system. Unfortunately, in many countries expensive anti‐PD drugs are either self‐funded or need expensive insurance. These pharmacoeconomic issues are important for the success of individualized therapy in PD.

### Nonmotor Subtypes of PD and Personalized Medicine

**Figure 5:**
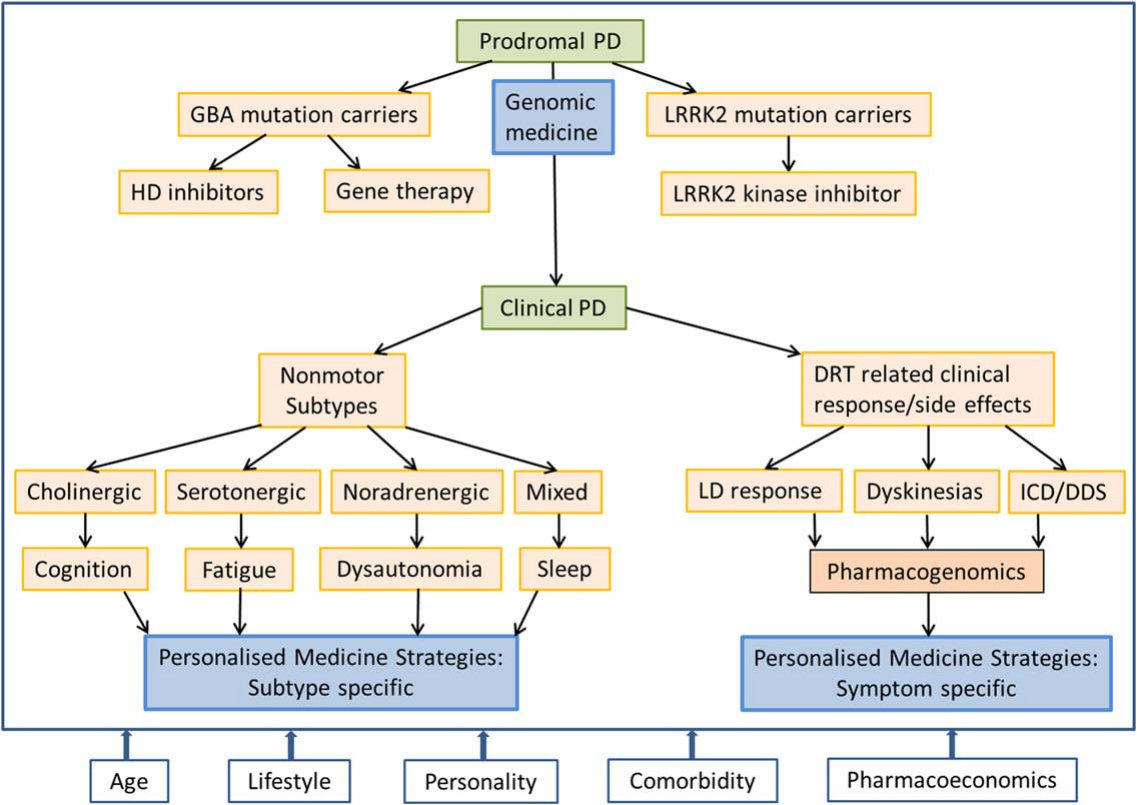
A summary of the various components of and strategies proposed to establish a comprehensive and holistic personalized medicine strategy.

The concept of nonmotor subtypes is based on the biomarker‐driven identification of phenotypes comprising of cholinergic, noradrenergic, serotonergic, and mixed neurotransmitter dysfunction underpinned by dopamine deficiency.^1^, ^2^ The resulting clinical phenotypes are likely to have nonmotor symptoms ranging from cognitive to sleep (Table 5).^3^, ^4^ These findings have also been replicated by cluster analysis of de novo PD cases and individual cohort studies.^31^, ^32^ Personalized medicine in these subtypes involves the treatment of specific nonmotor symptoms and consideration of the nonmotor side effects (such as sudden onset of sleep) of dopaminergic drugs. This can be achieved by a multimodal approach with imaging, genetic, pharmacogenetic biomarkers resulting in a subtype‐specific treatment strategy (Fig. 5). Proposed strategies are summarized in Table 5. In addition, future imaging may help stratify treatment in patients susceptible to DRT‐related side effects. Imaging showing abnormal dopamine turnover or release (eg, in ventral striatum) may imply susceptibility to levodopa‐induced dyskinesias or ICD and help develop tailored therapy.

**Table 5:** Nonmotor subtypes in Parkinson's disease and proposed personalized medicine–based treatment paradigm

Neurotransmitter dysfunction based syndromes in PD	Proposed clinical NMS subtypes	Clinical implications	Biomarkers			Proposed personalized medicine strategy
			Imaging	Genetic	Clinical	
Cholinergic	Park‐cognitive (including MCI, Apathy, RBD, postural instability) Apathy could be cholinergic and dopaminergic	Patients at high risk of dementia, cognitive dysfunction, falls	Brain: PET scans with *N*‐[11C]‐methyl‐4‐piperidyl acetate acetylcholinesterase (AChE): ↓ activity 33 Gut: [11C]donepezil PET (colon, intestine, pancreas): ↓ activity^34^	Microtubule‐associated protein Tau (MAPT) gene (H1/H2 genotype)^35^	↓Semantic fluency Difficulty in pentagon copying 35	Cognitive decline: Counseling regarding lifestyle Forward planning Combining DRT and cholinesterase inhibitor therapy Cognitive training
Noradrenergic	Park ‐autonomic	Risk of aspiration pneumonia, weight loss, poor oral DRT absorption	Heart: Cardiac Metaiodobenzylguanidine (MIBG) scanning^36^	No robust markers	Upper and lower gastrointestinal dysfunction: Delayed gastric emptying Constipation Symptomatic postural hypotension	Consider nonoral therapies as a 1st option for DRT Nutritional and dietary advice Probiotic supplementation Supplement DRT with conventional management of postural hypotension Nonmotor fall prevention strategy (extra caution if patient also has osteoporosis)
Serotonergic	Park ‐fatigue	Clinical picture similar to chronic fatigue syndrome	Brain: [^11^C]DASB PET abnormality in limbic striatum an insula^37^	No robust marker	Abnormal fatigue scale severity score	Combining DRT with serotonergic agents (receptor agonists)
Mixed neurotransmitter dysfunction	Park‐ sleep	Somnolence, sudden onset of sleep “attacks”	Brain: [^11^C]DASB PET abnormality in raphe area^38^	DRD2 and DRD4 (both linked to “sleep attacks,” HCRT (preprohypocretin)^18^, ^19^	Clinical picture similar to narcolepsy with or without cataplexy Co‐occurrence with RBD^39^	Avoid dopamine D3 receptor active agonists (may precipitate sudden onset of sleep) Consider alerting agents Early advice regarding lifestyle (avoid driving, working with heavy machinery, swimming alone) ? role of serotonergic agents (receptor agonists)

NMS, nonmotor symptoms; MCI, mild cognitive impairment; PET, positron emission tomography; DASB, 3‐amino‐4‐2‐dimethylaminomethylphenylsulfanyl‐benzonitrile; DRD, dopamine receptor; DRT, dopamine replacement therapy; RBD, rapid eye movement behavior disorder.

## Conclusions

Contrary to the common perception that personalized medicine is completely based on a genetic approach, we feel a holistic strategy spanning genes, clinical subtypes, personality, lifestyle, aging, and comorbidities constitute true personalized medicine. Enriching the phenotypic expression of PD with a multimodal clinical‐ and biomarker‐based approach may be the best way to address individualized treatment to achieve better clinical effect (Fig. 5). However, the complex nature of PD coupled with clinical phenotypic heterogeneity presents major challenges for formulating successful personalized medicine. Further research is therefore urgently needed to evaluate the best ways to deliver state‐of‐the‐art personalized medicine in PD.

## Author Roles

1) Research project: A. Conception, B. Organization, C. Execution; 2) Statistical Analysis: A. Design, B. Execution, C. Review and Critique; 3) Manuscript: A. Writing of the first draft, B. Review and Critique.

N.T.: 1A, 1B, 1C, 3A, 3B

K.R.C.: 1A, 1B, 1C, 3A, 3B

## Full financial disclosures for the previous 12 months

K.R.C. reports consultant/expert witness with Britannia, AbbVie, Neuronova, Mundipharma, and Union Chimique Belge (UCB); grants/research support from Boehringer Ingelheim, GlaxoSmithKline (GSK) Pharamaceuticals, Britania Pharmaceuticals, AbbVie, UCB, Neuronova, Academic: European Union (EU), Parkinson's UK, National Institute of Health Research (NIHR), and Parkinson's disease non motor group (PDNMG); honoraria/speakers bureau with Boehringer Ingelheim, GSK Pharamaceuticals, AbbVie, Britannia, UCB, and Mundipharma; intellectual property rights for the Kings Parkinson's Pain Scale and PDSS 2; and royalty/patents from Oxford University Press and Fastfacts: Parkinson's Disease. N.T. has nothing to disclose.
